# Biography of Members of Massachusetts Dental Society

**Published:** 1914-10-15

**Authors:** 


BIOGRAPHY OF MEMBERS OF MASSACHUSETTS DENTAL SOCIETY. We have before us a neatly gotten up book of 224 pages, edited by Dr. Waldo E. Boardman, of Boston, in which he sketches in an interesting manner the lives and professional careers of the more prominent men who have contributed to the founding and development of the profession in that state. Many of the names have no personal relations outside of New England, but all are more or less known from their literary contributions. Some of course, have wide national and international reputation. The editor has included some names who are more generally known in other states, like W. H. Atkinson and J. H. McQuillan, but it seems that these men were big enough to have lent of their talents to the founding of dentistry even in New England. It is a worthy contribution to our literature, and it would be well if each state could have a similar volume for a future hall of fame, or as a source of information at least.

